# Evaluation of the Effect of Conversion of Nonvalvular Atrial Fibrillation to Sinus Rhythm on Cardiac Remodeling

**DOI:** 10.7759/cureus.60504

**Published:** 2024-05-17

**Authors:** Babak Payami, Nehzat Akiash, Mohammadreza Kiarsi, Amir Moradi, Mohammad Kheradmandpour, Somayeh Abbaspour

**Affiliations:** 1 Atherosclerosis Research Center, Ahvaz Jundishapur University of Medical Sciences, Ahvaz, IRN; 2 Department of Cardiology, Ahvaz Jundishapur University of Medical Sciences, Ahvaz, IRN

**Keywords:** cardiac remodeling, cardiac output, stroke volume, electrical cardioversion, atrial fibrillation

## Abstract

Background: Atrial fibrillation (AF) represents the most prevalent cardiac arrhythmia globally, with a significant burden on mortality and morbidity. While rhythm control strategies, particularly electrical cardioversion (EC), have gained traction in recent years, the precise impact of sinus rhythm (SR) restoration on cardiac reverse remodeling remains a subject of debate.

Methods: In this study, 23 AF patients underwent elective EC. AF diagnosis was made via ECG by a cardiologist, and candidates for cardioversion were selected by an electrophysiologist. Transthoracic echocardiography (TTE) by utilizing two-dimensional, three-dimensional, and tissue Doppler imaging modalities was performed before cardioversion. Patients who maintained SR after six months underwent a second TTE evaluation.

Results: SR was restored successfully in all 23 patients and 15 patients (65.2%) maintained SR after six months. SR group had significantly lower baseline cardiac output (CO) and indexed left ventricular end-systolic volume (LVESVi), and better European Heart Rhythm Association (EHRA) scores after six months. Within the SR group, patients exhibited significant changes in mitral regurgitation, tricuspid regurgitation, EHRA score, LVESVi, stroke volume, left ventricle ejection fraction, left ventricle global longitudinal strain, indexed minimum left atrial volume, left atrial emptying fraction, and left and right atrial diameters. Reduced CO was associated with AF recurrence. Receiver operating curve analysis revealed that CO value can predict six-month AF recurrence with a cut-off point of 2.3.

Conclusion: Our study underscores the beneficial effects of SR restoration on cardiac parameters in AF patients post EC. Notably, CO value emerged as a predictor of AF recurrence, emphasizing the importance of comprehensive assessments for predicting long-term outcomes.

## Introduction

Atrial fibrillation (AF) stands as the most prevalent cardiac arrhythmia globally, affecting over 46 million individuals worldwide [1], with an incidence rate that has increased by one-third in the last two decades. The projected burden is anticipated to escalate by more than 60% by 2050 [2]. This condition significantly increases mortality and morbidity as patients experience a higher risk of stroke, myocardial infarction, congestive heart failure, systemic thromboembolism, chronic kidney disease, peripheral artery disease, and dementia [3]. The relationship between cardiac dysfunction and AF is complex, with each capable of being both the cause and consequence of the other, exacerbating each other through a positive feedback loop [4].

While the historical approach to AF management centered on ventricular rate control, a shift in clinicians’ preference toward rhythm control has emerged in light of new targeted therapies [5]. Current guidelines advocate for a trial of rhythm control in all AF patients with left ventricular (LV) dysfunction [3]. Rhythm control not only contributes to symptom alleviation [6] but also demonstrates efficacy in reducing AF progression [7], hospitalization, and mortality [8]. Patient characteristics such as younger age, shorter AF duration, increased symptoms, smaller left atrial (LA) dimensions, more LV dysfunction, and higher grades of atrioventricular regurgitation delineate a subgroup that may particularly benefit from rhythm control [3]. Rhythm control is achievable through pharmacological or electrical cardioversion (EC), with the latter being a more efficient way of restoring sinus rhythm (SR) [9,10].

While the effects of SR restoration on left ventricular ejection fraction (LVEF), physical activity, and quality of life in AF patients have been previously reported [11,12], the exact impact of SR restoration on cardiac reverse remodeling is still a subject of debate. Controversial findings regarding functional and volumetric indices after EC have been reported, which were compared with the results of this article in the discussion section. Hence, this study aims to assess functional and volumetric changes in AF patients six months post cardioversion and compare echocardiographic characteristics of those who experienced AF recurrence with individuals maintaining SR after six months.

## Materials and methods

The present investigation was conducted prospectively on individuals afflicted with nonvalvular AF who underwent elective EC in Imam Khomeini and Golestan Hospitals, prominent tertiary healthcare institutes in Ahvaz, Iran, from June 2022 to May 2023. The study protocol was explained thoroughly to all enrolled subjects, and informed consent was obtained from them prior to the commencement of the study. The study was reviewed and approved by the Ethics Committee of Ahvaz Jundishapur University of Medical Sciences with the approval ID of IR.AJUMS.HGOLESTAN.REC.1401.172.

Study protocol

Inclusion criteria encompassed patients aged 18 years or older with nonvalvular AF initially recruited into the study. AF was diagnosed by a cardiologist based on the patient’s ECG and candidates for cardioversion were selected by an electrophysiologist. Patients with a prior history of EC, organic valvular diseases, prosthetic cardiac valve, contraindications for anticoagulant therapy, a history of psychiatric disorders, inability to attend follow-up visits, or improper echocardiographic images were excluded from the study. Demographic characteristics of study participants were collected through face-to-face interviews and reviewing medical records. Transthoracic echocardiography (TTE) studies were performed by trained echocardiologists on the day preceding cardioversion. A cardiac electrophysiologist performed a thorough examination six months post cardioversion, and patients who had maintained SR underwent another TTE to assess cardiac changes.

Electrical cardioversion

All patients received proper anticoagulants from three weeks before cardioversion to four weeks after the intervention. EC was performed under general sedation, using IV midazolam under the supervision of an anesthesiologist. Electric shock was delivered by a biphasic defibrillator with electrodes placed in an anterior-posterior position. The initial delivered energy was 200 J. If the first attempt was unsuccessful, a second attempt by adding 100 J was tried. A successful cardioversion was defined as achieving and maintaining SR for at least 24 hours. Based on the American Heart Association (AHA) recommendations, patients received antiarrhythmic drugs after discharge [3].

Echocardiographic assessments

Echocardiographic assessments were conducted employing a comprehensive approach, incorporating two-dimensional (2D) echocardiography, three-dimensional (3D) echocardiography, and tissue Doppler imaging (TDI) modalities with speckle tracking analysis for chamber qualification.

TTE was performed utilizing a Vivid E9 ultrasound machine (GE Vingmed Ultrasound, Horten, Norway) equipped with a GE 4V-D (1.5-4 MHz) probe for 3D speckle tracking echocardiography (3D-STE) and GE M5s (1.4-4.6 MHz) probe for 2D echocardiography. The acquisition of volumetric echocardiographic data adhered to the American Society of Echocardiography guidelines. Image acquisition was performed in at least three consecutive cardiac cycles in patients with SR and five in patients with AF to ensure capturing interbeat variability [13].

2D echocardiography

LA area was measured from two- and four-chamber views by tracing the endocardial border. LA volume (LAV) calculations were performed using the biplane area-length method formula [13]: LAV = (0.85 × A1 × A2)/L, in which A1 and A2 are LA area in two- and four-chamber views, and L is the shortest of the two long axes measured in the apical two-and four-chamber view. LA emptying fraction (LAEF) was also calculated using the following formula: LAEF (%) = ((LAVmax - LAVmin)/LAVmax) × 100.

3D echocardiography

Regarding the assessment of LV using 3D-STE, after data acquisition, the 4D Auto LVQ software (EchoPAC BT13, GE Vingmed Ultrasound, Horten, Norway) was employed for volume analysis, enabling the determination of left ventricular end-diastolic volume (LVEDV), left ventricular end-systolic volume (LVESV), and LVEF. Three anatomical landmarks, comprising two points at the mitral annulus edges and one at the apex, were identified in both end-diastolic and end-systolic frames across each apical plane. The software automatically delineated the LV endocardial border in a 3D model, with manual adjustments applied when necessary due to inadequate automatic delineation. Additionally, left ventricular global longitudinal strain (LVGLS) was quantified through a second epicardial tracking, and LV strain was evaluated by automatic delineation of the region of interest. The software automatically ascertained LVGLS and borders, with manual adjustments in case of inaccuracies.

Statistical analysis

Descriptive statistics are used to present the data, with frequencies and percentages for categorical variables and means and standard deviations for continuous variables. The normality of the data distribution and the equality of variances were assessed using the Kolmogorov-Smirnov and Levene's tests, respectively. Independent sample’s T-test or Wilcoxon test (in case of not-normally distributed data) were used to compare continuous variables whereas chi-square or Fisher’s exact test was used to compare categorical data between groups. Logistic regression analysis was applied to explore the association of different variables with AF recurrence. Additionally, receiver operating characteristic (ROC) curve analysis was conducted to determine the optimal cut-off point for predictors of AF recurrence. IBM SPSS Statistics for Windows version 27 (IBM Corp., Armonk, NY) was used, and statistical significance was defined as P-values less than 0.05.

## Results

A total of 23 patients, with a mean age of 56.9 ± 13.1 years, participated in the study. The mean duration from initial AF diagnosis to cardioversion was 12.3 ± 18.3 months, and the most prevalent comorbidity among study participants was hypertension (47.8%), followed by diabetes mellitus (30.1%). All patients had successful SR restoration, and no complications occurred during the intervention. After a six-month follow-up, 15 patients (65.2%) maintained sinus rhythm (SR group), while eight patients (34.8%) had recurrent AF (AF group) (Table 1).

**Table 1:** Demographic and clinical characteristics of SR and AF groups. SR, sinus rhythm; AF, atrial fibrillation; IHD, ischemic heart disease; CVA, cerebrovascular accident; MR, mitral regurgitation; TR, tricuspid regurgitation; EHRA score, European Heart Rhythm Association score of atrial fibrillations; CHA_2_DS_2_-VASc score, congestive heart failure, hypertension, age ≥ 75 years, diabetes mellitus, stroke or transient ischemic attack, vascular disease, age 65 to 74 years, sex category. * P-value < 0.05; ** p-value < 0.001. Descriptive statistics are used to present the data, with frequencies and percentages for categorical variables and means and standard deviations for continuous variables.

Variable		Total (N = 23)	SR group	AF group	P-value
Age (years) (mean ± SD)		56.9 ± 13.1	56.2 ± 14.0	58.2 ± 11.6	0.728
Time from first diagnosis to cardioversion (months) (mean ± SD)		12.30 ± 18.35	16.27 ± 21.69	4.88 ± 4.54	0.068
Gender, N (%)	Female	11 (47.8%)	5 (33.3%)	6 (26.1%)	0.089
	Male	12 (52.2%)	10 (66.7%)	2 (8.7%)	
History of IHD, N (%)		2 (8.7%)	2 (13.3%)	0 (0.0%)	0.526
Diabetes mellitus, N (%)		7 (30.1%)	6 (40.0%)	1 (12.5%)	0.350
Hypertension, N (%)		11 (47.8%)	8 (53.3%)	3 (37.5%)	0.667
Dyslipidemia, N (%)		4 (17.4%)	2 (13.3%)	2 (25%)	0.589
History of CVA, N (%)		0 (0.0%)	0 (0.0%)	0 (0.0%)	-
Smoke, N (%)		3 (13.0%)	3 (20.0%)	0 (0.0%)	0.526
Mitral regurgitation, N (%)	No MR	1 (4.3%)	1 (6.7%)	0 (0.0%)	0.478
	Mild	6 (26.1%)	2 (13.3%)	4 (50%)	
	Moderate	12 (52.2%)	10 (66.7%)	2 (25%)	
	Severe	2 (8.7%)	2 (13.3%)	0 (0.0%)	
	Critical	2 (8.7%)	2 (13.3%)	2 (25%)	
Tricuspid regurgitation, N (%)	No TR	1 (4.3%)	1 (6.7%)	0 (0.0%)	0.605
	Mild	6 (26.1%)	3 (20.0%)	3 (37.5%)	
	Moderate	10 (43.5%)	8 (53.3%)	2 (25%)	
	Severe	4 (17.4%)	2 (13.3%)	2 (25%)	
	Critical	2 (8.7%)	1 (6.7%)	1 (12.5%)	
EHRA score before cardioversion, N (%)	I	1 (4.3%)	1 (6.7%)	0 (0.0%)	0.179
	II	17 (73.9%)	9 (60%)	8 (100%)	
	III	4 (17.4%)	4 (26.6%)	0 (0.0%)	
	IV	1 (4.3%)	1 (6.7%)	0 (0.0%)	
EHRA score six months after cardioversion, N (%)	I	15 (65.2%)	15 (100.0%)	0 (0.0%)	**<0.001
	II	3 (13.0%)	0 (0.0%)	3 (37.5%)	
	III	4 (17.4%)	0 (0.0%)	4 (50.0%)	
	IV	1 (4.4%)	0 (0.0%)	1 (12.5%)	
CHA_2_DS_2_-VASc score, N (%)	0	4 (17.4%)	3 (20%)	1 (12.5%)	0.311
	1	4 (17.4%)	2 (13.3%)	2 (25%)	
	≥2	15 (65.2%)	10 (66.7%)	5 (62.5%)	
			SR group (baseline)	SR group (after 6 months)	
Mitral regurgitation, N (%)	No MR		1 (6.7%)	2 (13.3%)	*0.002
	Mild		2 (13.3%)	11 (73.4%)	
	Moderate		10 (66.7%)	2 (13.3%)	
	Severe		2 (13.3%)	0 (0.0%)	
	Critical		0 (0.0%)	0 (0.0%)	
Tricuspid regurgitation, N (%)	No TR		1 (6.7%)	1 (6.7%)	*0.002
	Mild		3 (20.0%)	12 (80.0%)	
	Moderate		8 (53.3%)	2 (13.3%)	
	Severe		2 (13.3%)	0 (0.0%)	
	Critical		1 (6.7%)	0 (0.0%)	
EHRA score, N (%)	I		1 (6.7%)	15 (100.0%)	**<0.001
	II		9 (60%)	0 (0.0%)	
	III		4 (26.6%)	0 (0.0%)	
	IV		1 (6.7%)	0 (0.0%)	

Comparison of demographic and clinical characteristics of SR and AF groups

Upon dividing participants into SR and AF groups based on their rhythm six months after cardioversion, no statistically significant differences were observed in age, gender, past medical history, baseline mitral or tricuspid regurgitation severity, and baseline EHRA score (all p-values > 0.05). However, a significant difference was noted between SR and AF groups concerning patients’ symptom severity six months post cardioversion (p < 0.001) (Table 1).

Comparison of echocardiographic findings of SR and AF groups

**Figure 1:**
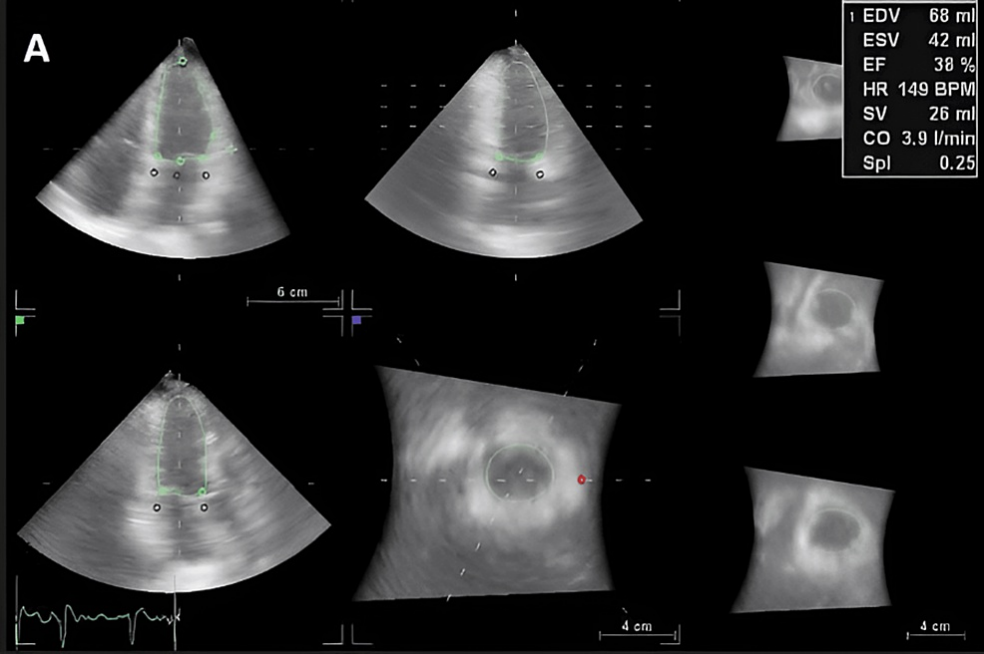
Evaluation of LV volumes, SV, and CO by 4D LVQ method before AF conversion. LV, left ventricle; SV, stroke volume; CO, cardiac output; AF, atrial fibrillation.

**Figure 2:**
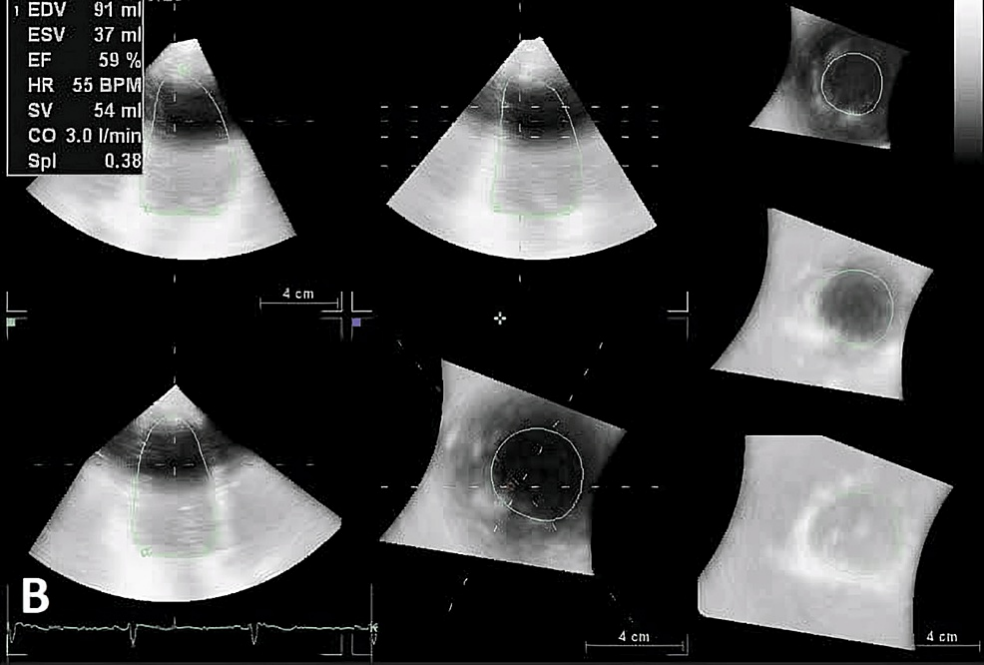
Evaluation of LV volume, SV, and CO by 4D LVQ method after AF conversion. LV, left ventricle; SV, stroke volume; CO, cardiac output; AF, atrial fibrillation.

**Figure 3:**
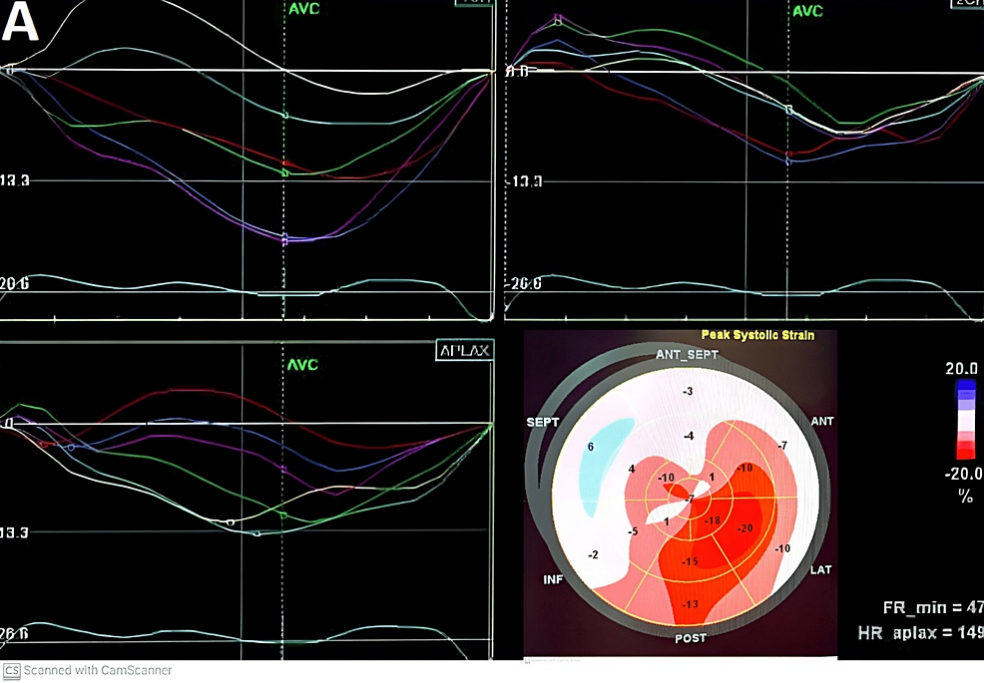
Evaluation of global longitudinal strain (GLS) before atrial fibrillation conversion.

**Figure 4:**
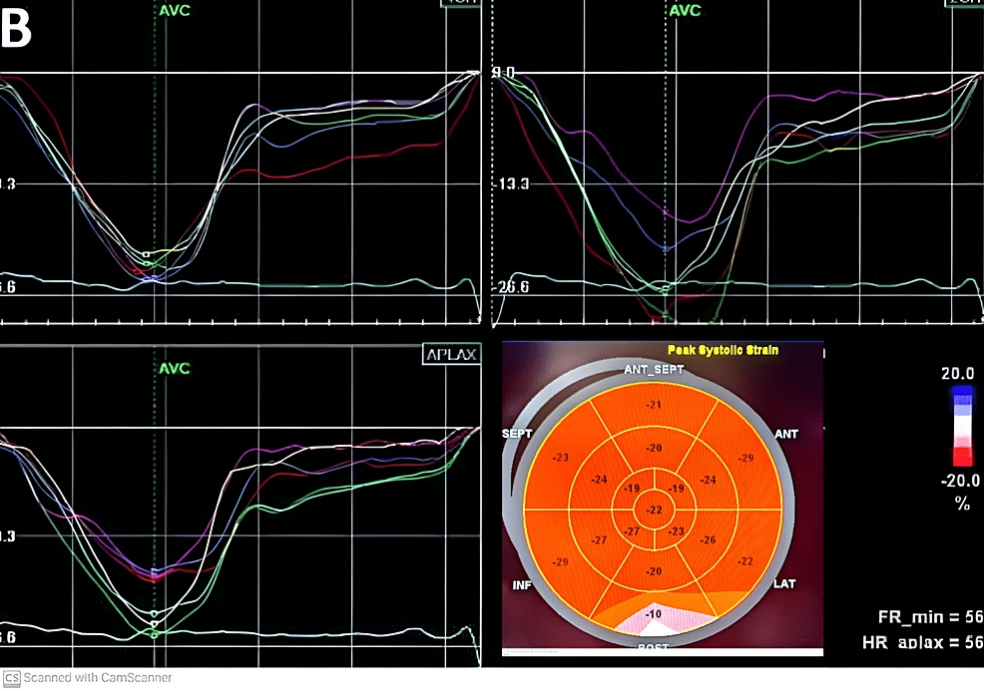
Evaluation of global longitudinal strain (GLS) after atrial fibrillation conversion.

Significant differences were observed between SR and AF groups in terms of cardiac output (CO) obtained by 2D echocardiography (p = 0.027) and indexed left ventricular end-systolic volume (LVESVi) obtained by 3D speckle tracking echocardiography (3D-STE) (p = 0.042). However, no statistically significant differences were found in indexed left ventricle end-diastolic volume (LVEDVi) by 2D and 3D-STE, LVESVi by 2D echocardiography, stroke volume (SV), left ventricle ejection fraction (LVEF), CO by 3D-STE, left ventricle global longitudinal strain (LVGLS), left atrial (LA) assessments (including LA diameter, length, and area, indexed left atrial volume (LAVi) minimum and maximum, and LAEF), and right heart assessments (including right atrial (RA) diameter, right ventricle diameter, tricuspid annular plane systolic excursion (TAPSE), and tricuspid regurgitation gradient (TRG)) (all p-values > 0.05) (Table 2 and Figures 1-4).

**Table 2:** Echocardiographic findings of SR and AF groups. SR, sinus rhythm; AF, atrial fibrillation; LV, left ventricle; LVEDVi, indexed left ventricle end-diastolic volume; LVESVi, indexed left ventricle end-systolic volume; LVEF; left ventricle ejection fraction; LA, left atrium; LAVi; indexed left atrial volume; LAEF, left atrial emptying fraction; RA, right atrium; RV, right ventricle; TAPSE, tricuspid annular plane systolic excursion; GLS, global longitudinal strain; TRG, tricuspid regurgitation gradient; MV, mitral valve; TV, tricuspid valve. * P-value < 0.05; ** p-value < 0.001. Descriptive statistics are used to present the data, with frequencies and percentages for categorical variables and means and standard deviations for continuous variables.

Parameters	View		AF group		SR group (baseline)		SR group (after 6 months)	P-value			
								AF vs. SR	SR (baseline vs. after 6 months)		
2D echocardiography											
LVEDVi (mL/m^2^), (mean ± SD)	2-chamber		46.67 ± 21.25		47.92 ± 24.71		47.12 ± 23.82	0.905		0.691	
	4-chamber		52.57 ± 18.85		52.52 ± 20.10		52.78 ± 15.78	0.996		0.733	
	Biplane		57.22 ± 42.12		50.44 ± 21.81		47.46 ± 18.87	0.613		0.798	
LVESVi (mL/m^2^), (mean ± SD)	2-chamber		29.96 ± 21.29		31.52 ± 20.60		22.66 ± 16.68	0.866		0.079	
	4-chamber		31.50 ± 19.83		34.13 ± 17.05		24.82 ± 11.05	0.742		0.005	
	Biplane		37.83 ± 39.69		32.22 ± 18.41		22.87 ± 11.55	0.645		0.005	
Stroke volume (mL), (mean ± SD)	2-chamber		29.50 ± 8.60		34.60 ± 10.88		47.13 ± 18.48	0.266		0.038	
	4-chamber		37.50 ± 12.28		37.33 ± 17.91		55.53 ± 16.93	0.982		*0.001	
	Biplane		32.88 ± 8.62		35.00 ± 12.91		49.47 ± 17.36	0.681		**0.009	
Cardiac output (L/min), (mean ± SD)	2-chamber		2.35 ± 0.82		3.37 ± 1.17		3.25 ± 1.42	0.041		0.529	
	4-chamber		3.16 ± 1.20		3.68 ± 1.85		3.79 ± 2.57	0.480		0.730	
	Biplane		2.48 ± 0.94		3.70 ± 1.27		3.57 ± 1.95	0.027		0.362	
LVEF (%), (mean ± SD)	2-chamber		40.00 ± 13.72		38.80 ± 12.90		54.73 ± 9.73	0.837		*0.002	
	4-chamber		43.63 ± 16.63		37.20 ± 12.81		53.93 ± 10.01	0.313		**<0.001	
	Biplane		41.13 ± 15.61		39.20 ± 11.09		54.40 ± 8.63	0.734		**<0.001	
LA diameter (cm), (mean ± SD)			3.83 ± 0.66		4.18 ± 0.57		3.68 ± 0.32	0.211		*0.001	
LA length (cm), (mean ± SD)	2-chamber		5.96 ± 0.70		5.72 ± 0.65		5.79 ± 0.68	0.420		0.529	
	4-chamber		6.52 ± 0.71		6.10 ± 0.72		5.88 ± 0.59	0.202		0.077	
LA area (cm^2^), (mean ± SD)	2-chamber		24.67 ± 4.39		22.70 ± 3.80		20.72 ± 3.50	0.275		0.083	
	4-chamber		23.52 ± 4.36		22.72 ± 4.61		20.96 ± 3.75	0.692		0.198	
LAVi min (mL/m^2^), (mean ± SD)			33.36 ± 11.97		28.35 ± 9.39		18.27 ± 6.24	0.280		**<0.001	
LAVi max (mL/m^2^), (mean ± SD)			41.65 ± 14.64		34.62 ± 9.23		32.06 ± 9.62	0.171		0.433	
LAEF (%), (mean ± SD)			32.92 ± 12.41		21.44 ± 15.52		41.80 ± 17.42	0.086		*0.002	
MV annulus diameter (cm), (mean ± SD)			2.93 ± 0.41		2.98 ± 0.32		2.82 ± 0.30	0.790		*0.013	
TV annulus diameter (cm), (mean ± SD)			3.16 ± 0.46		3.07 ± 0.63		2.88 ± 0.41	0.730		0.091	
RA diameter, (mean ± SD)			3.65 ± 0.68		3.84 ± 0.70		3.46 ± 0.68	0.528		*0.005	
RV diameter (cm), (mean ± SD)			2.95 ± 0.41		3.22 ± 0.48		3.12 ± 0.037	0.189		0.163	
TAPSE (mm), (mean ± SD)			18.63 ± 4.30		19.13 ± 4.06		20.73 ± 2.76	0.782		0.166	
3D echocardiography											
LVEDVi (mL/m^2^), (mean ± SD)			46.78 ± 13.35		49.70 ± 14.98		53.41 ± 12.93	0.676		0.347	
LVESVi (mL/m^2^), (mean ± SD)			36.10 ± 25.49		29.67 ± 12.90		25.45 ± 9.59	0.042		*0.041	
Stroke volume (mL), (mean ± SD)			32.29 ± 10.98		38.33 ± 15.28		53.43 ± 12.32	0.374		*0.041	
Cardiac output (L/min), (mean ± SD)			3.05 ± 1.40		3.90 ± 1.97		3.63 ± 0.92	0.332		0.790	
LVEF (%), (mean ± SD)			38.71 ± 15.48		41.50 ± 8.70		53.27 ± 8.44	0.619		*0.002	
LVGLS (%), (mean ± SD)	2-chamber		-14.05 ± 3.58		-10.56 ± 5.13		-17.06 ± 5.84	0.103		*0.003	
	3-chamber		-13.63 ± 4.93		-8.90 ± 3.31		-16.56 ± 5.93	0.012		*0.001	
	4-chamber		-12.88 ± 4.81		-10.24 ± 4.65		-17.81 ± 4.93	0.213		**<0.001	
	Average		-13.52 ± 4.11		-9.88 ± 3.96		-17.07 ± 5.39	0.051		*0.001	
Tissue Doppler imaging											
e’ septal (cm/s), (mean ± SD)			7.63 ± 4.34		8.27 ± 3.80		10.00 ± 2.61	0.717		*0.016	
e’ lateral (cm/s), (mean ± SD)			11.00 ± 4.95		9.87 ± 4.34		11.53 ± 3.18	0.576		0.075	
TRG (mmHg), (mean ± SD)			25.50 ± 4.92		22.07 ± 5.10		19.13 ± 5.47	0.135		0.078	

Changes in the SR group

Patients in the SR group demonstrated significant improvements in symptom severity and function (p < 0.001), mitral regurgitation severity (p < 0.002), and tricuspid regurgitation severity (p < 0.002) six months after cardioversion (Table 1).

Echocardiographic changes in the SR group

**Figure 5:**
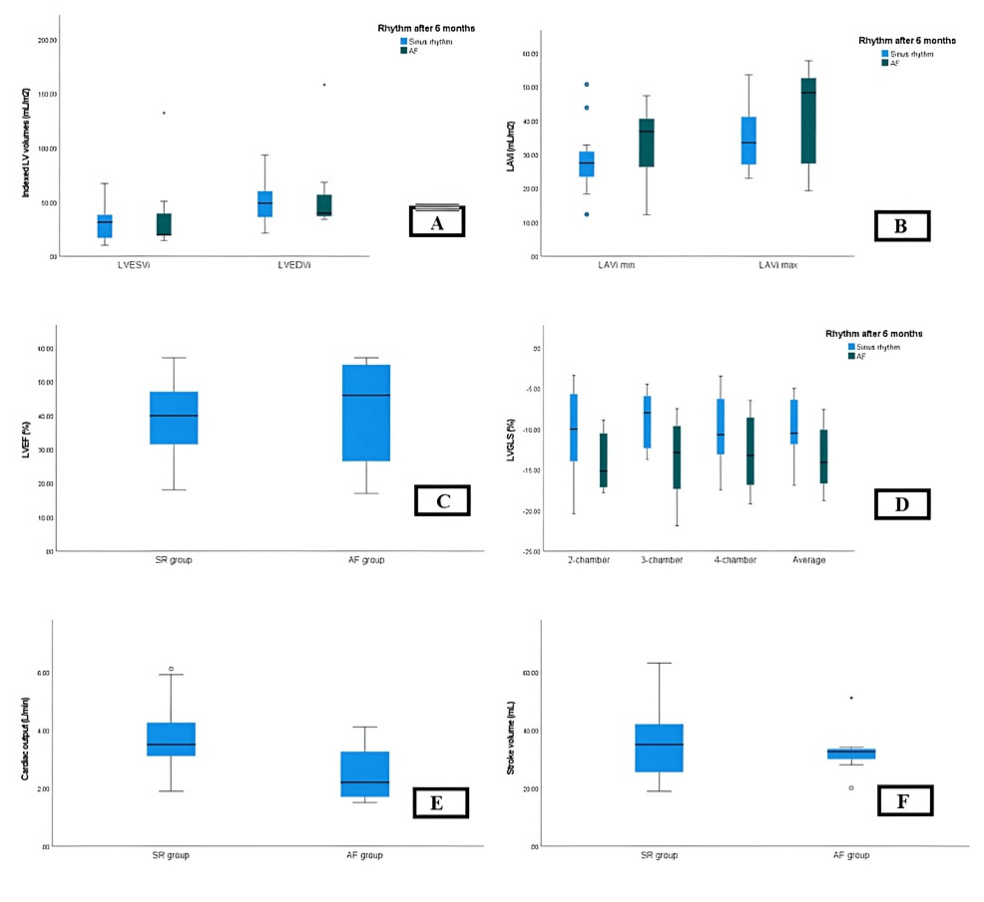
Comparison of baseline echocardiographic measurements of study participants stratified by rhythm at six-month follow-up. A: No statistically significant differences were observed in LVESVi and LVEDVi. B: No statistically significant differences were observed in maximum and minimum LAVi. C: No statistically significant differences were observed in LVEF. D: Significant differences were observed between SR and AF groups in terms of indexed left ventricular end-systolic volume (LVESVi) obtained by 3D speckle tracking echocardiography (3D-STE) (p = 0.042). E: Significant differences were observed between SR and AF groups in terms of cardiac output (CO) obtained by 2D echocardiography (p = 0.027) F: No statistically significant differences were found in stroke volume (SV). SR, sinus rhythm; AF, atrial fibrillation; LVEDVi, indexed left ventricle end-diastolic volume; LVESVi, indexed left ventricle end-systolic volume; LVEF; left ventricle ejection fraction; LAVi; indexed left atrial volume; LVGLS, left ventricle global longitudinal strain.

**Figure 6:**
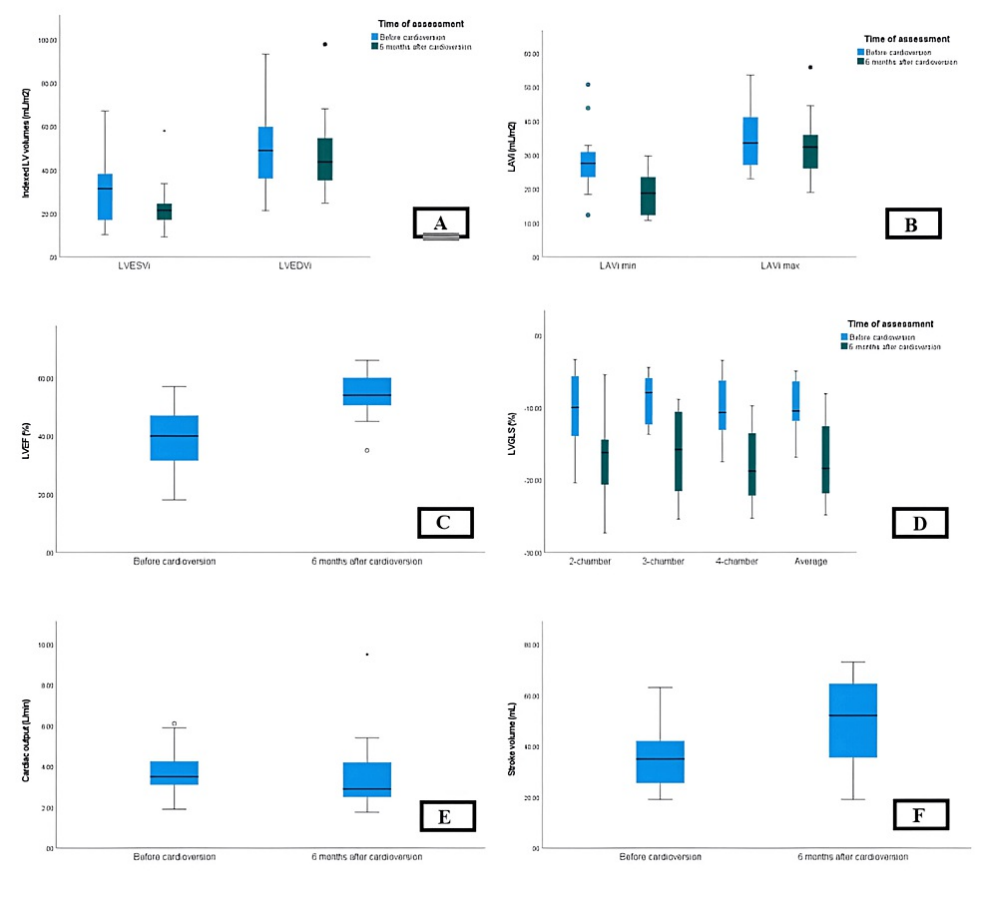
Comparison between baseline and post-cardioversion echocardiographic measurements of patients with sinus rhythm at six-month follow-up. A: Significant reductions were observed in LVESVi (both by biplane method, p < 0.005, and 3D-STE, p = 0.041). B: Significant reductions were observed in LAVi min (p = 0.001). C: Significant increases were observed in LVEF (both by biplane method, p < 0.001, and 3D-STE, p = 0.002). D: Significant increases were observed in LVGLS (p = 0.001). E: No statistically significant differences were observed in CO. F: Significant increases in SV (both by biplane method, p = 0.009, and 3D-STE, p = 0.041). LV, left ventricle; LVEDVi, indexed left ventricle end-diastolic volume; LVESVi, indexed left ventricle end-systolic volume; LVEF; left ventricle ejection fraction; LAVi; indexed left atrial volume; LVGLS, left ventricle global longitudinal strain; 3D-STE, 3D speckle tracking echocardiography; SV, stroke volume; CO, cardiac output.

Significant reductions were observed in LVESVi (both by biplane method, p < 0.005, and 3D-STE, p = 0.041), LA diameter (p = 0.001), LAVi minimum (p = 0.001), mitral valve annulus diameter (p = 0.013), and RA diameter (p = 0.005) in the SR group six months after cardioversion. Additionally, SR patients exhibited significant increases in SV (both by biplane method, p = 0.009, and 3D-STE, p = 0.041), LVEF (both by biplane method, p < 0.001, and 3D-STE, p = 0.002), LVGLS (p = 0.001), LAEF (p = 0.002), and e’ septal (p = 0.016). However, other echocardiographic measurements, including LVEDVi, CO, LA length and area, LAVi max, tricuspid valve annulus diameter, e’ lateral, RV diameter, TAPSE, and TRG (all p-values > 0.05) (Table 2 and Figures 5, 6).

Predictors of recurrent AF

**Figure 7:**
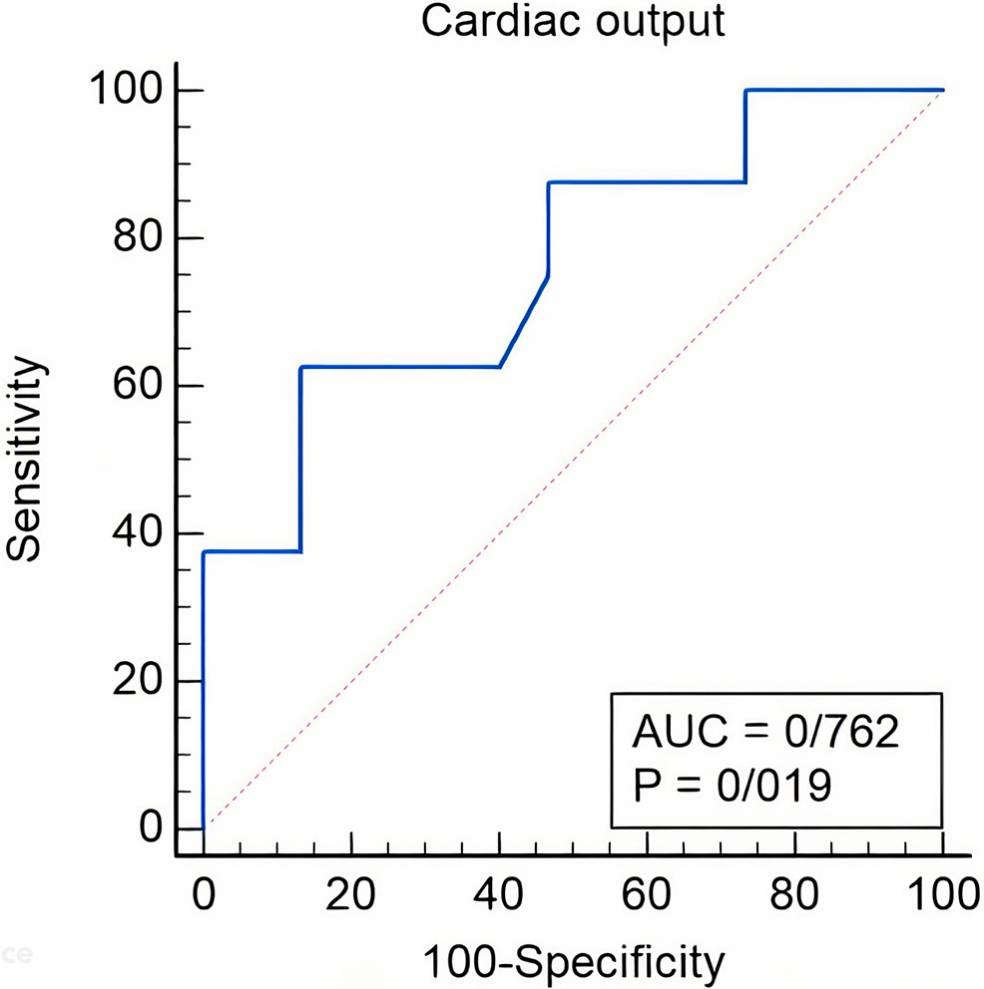
ROC curve to determine the cut-off value of cardiac output for prediction of AF recurrence at six months after electrical cardioversion. ROC, receiver operating characteristic; AUC, area under the curve; AF, atrial fibrillation.

Cardiac output was significantly associated with AF recurrence (OR: 0.33; 95% CI: 0.11-0.98; p = 0.047) (Table 3). Receiver operating curve analysis revealed that CO accurately predicts AF recurrence six months post-cardioversion, with an optimal cut-off point of 2.3 (area under the curve: 0.762; 95% CI: 0.541-0.913; p = 0.019; sensitivity 62.50%; specificity: 86.67%) (Table 3 and Figure 7).

**Table 3:** Factors determining AF recurrence at six-month follow-up – univariate regression analysis of demographic and echocardiographic data. DM, diabetes mellitus; HTN, hypertension; HLP, hyperlipidemia; MR, mitral regurgitation; TR, tricuspid regurgitation; LVEDVi, indexed left ventricle end-diastolic volume; LVESVi, indexed left ventricle end-systolic volume; LVEF, left ventricle ejection fraction; LVGLS, left ventricle global longitudinal strain; LAEF, left atrial emptying fraction; LAVi, indexed left atrial volume; TAPSE, tricuspid annular plane systolic excursion; CHA_2_DS_2_-VASc score, congestive heart failure, hypertension, age ≥ 75 years, diabetes mellitus, stroke or transient ischemic attack, vascular disease, age 65 to 74 years, sex category. * P-value < 0.05. To explore the association of different variables with atrial fibrillation (AF) recurrence, logistic regression analysis was applied.

Variable		OR	95% CI		P-value
			Lower	Upper	
Age		1.01	0.94	1.08	0.714
Male gender		0.16	0.02	1.14	0.068
DM		4.00	0.37	42.17	0.249
HTN		1.90	0.33	11.00	0.472
HLP		2.167	0.244	19.27	0.488
CHA_2_DS_2_-VASc score	0	Ref	-	-	-
	1	3.00	0.150	59.89	0.472
	≥2	1.50	0.12	18.36	0.751
Moderate/severe MR		4.00	0.61	26.12	0.148
Moderate/severe TR		1.65	0.26	10.31	0.592
LVEDVi		1.00	0.97	1.03	0.599
LVESVi		1.00	0.97	1.04	0.631
Stroke volume		0.98	0.90	1.06	0.666
Cardiac output		0.33	0.11	0.98	*0.047
LVEF		1.01	0.94	1.08	0.720
LVGLS		1.25	0.98	1.59	0.064
LAEF		1.05	0.98	1.12	0.101
LAVi min		1.05	0.96	1.15	0.271
LAVi max		1.05	0.97	1.14	0.169
TAPSE		0.96	0.77	1.20	0.770
e’ septal		0.95	0.76	1.20	0.702
e’ lateral		1.06	0.87	1.28	0.558

## Discussion

In the current study, we compared baseline echocardiographic measurements of AF patients who underwent electric cardioversion and had recurrent AF with patients who maintained SR after six months (SR group). Additionally, we investigated the echocardiographic changes in the SR group after six months. Our analysis revealed notable distinctions between baseline CO, LVESVi, and six-month EHRA scores between SR and AF groups. Furthermore, the SR group exhibited significant improvements in EHRA score, mitral and tricuspid regurgitation severity, LVESVi, LVEF, LVGLS, LAEF, LAVi minimum, SV, LA and RA diameter, mitral valve annulus diameter, and e’ septal.

AF, as the most prevalent sustained cardiac arrhythmia, presents numerous challenges. AF remains a substantial global health concern despite a recent decline in prevalence and incidence [14]. The intricate relationship between AF and the mitral and tricuspid valves involves reciprocal exacerbation through various mechanisms. Furthermore, AF induces cardiac remodeling over time, contributing to valve dysfunction and impacting patients' functional capacity. The restoration of SR has been identified as a pivotal factor in improving patients’ quality of life. Consistent with existing literature [15-17], we noted significant enhancements in valvular dysfunction severity and patients' functional capacity.

The impact of AF on ventricular function is well-established, with AF being capable of reducing LV function through various mechanisms, such as LV dilation and systolic dysfunction [18]. Consistent with previous investigations [19-21], our study identified a significant improvement in LVEF following cardioversion. Additionally, LVGLS, a novel index of ventricular function, was observed to be improved after the cardioversion, which was also previously documented [22]. This improvement can be attributed to the cessation of tachycardic episodes, Frank-Starling mechanisms, and the potential for reverse remodeling.

While AF induces cardiac remodeling over time, the impact of restoring the SR on echocardiographic atrial and ventricular volume indices remains a topic of debate. Baseline LVESVi was significantly different between SR and AF groups in 3D echocardiography. Moreover, LVESVi significantly decreased after six months in the SR group. However, no notable difference was observed in baseline LVEDVi between the SR and AF groups and within the SR group six months post cardioversion. The literature presents conflicting findings on this matter. Karlsson et al. reported no changes in LVEDVi, while LVESVi significantly decreased in AF patients successfully maintaining SR after four weeks [23]. In Soulat-Dufour et al.’s study, patients with SR demonstrated significant changes in LVEDVi but not LVESVi after a 12-month follow-up [24]. In another study by Zimmermann et al., no notable differences were present regarding LVESV and LVEDV between patients with recurrent AF and those with SR in follow-up. Furthermore, SR patients demonstrated significant changes in LVEDVi but not LVESVi in the follow-up period [25]. The present discrepancies highlight the necessity for more extensive studies incorporating novel techniques to draw definitive conclusions regarding the effects of cardioversion on LV volumetric indices.

AF can cause several hemodynamic changes, among which alterations in cardiac output stand out. The alteration of CO in AF patients can occur through various mechanisms, including ventricular diastolic dysfunction, diminished left atrial contraction, and heart rate variability. Our study observed a notable CO difference between patients with AF and those maintaining SR after six months. This finding aligns with a study by Klavebäck et al. involving 44 AF patients undergoing electrical cardioversion, where no significant difference was observed in baseline CO of patients with recurrent AF compared with the SR group; however, the SR group had experienced considerable improvement in CO [26]. To the best of our knowledge, limited research has explored the effects of cardiac output in AF patients, and more extensive studies are warranted to assess the association of baseline CO with successful EC in this population.

SV demonstrated significant improvement in the SR group after six months, whereas CO did not exhibit a similar change. Similar to our findings, Karlsson et al. reported a substantial change in SV but not CO in AF patients after successful EC [23]. This observation may be attributed to the high heart rates of AF patients, as the precise assessment of stroke volume might be challenging due to the beat-to-beat variability.

LAVi and LAEF are widely recognized indices of left atrial function. Both indices have been reported to be improved after successful cardioversion [24,25]. LAVi, in particular, has consistently differed between patients with recurrent AF and those maintaining SR [4,27], serving as a predictor of AF recurrence [27,28]. Additionally, left atrial enlargement, a well-known consequence of AF [29], has been shown to improve with the restoration of SR in AF patients [30]. The Improvement of LAVi can be a consequence of breaking the vicious cycle of AF-cardiac remodeling and restoring the mechanical function of the atrium, ultimately leading to atrial reverse remodeling. In the present study, we observed a significant improvement in the LA diameter and LAVi minimum among patients maintaining SR after six months. However, no difference was observed in the baseline measurements of LAVi between the SR and AF groups. The observed discrepancy between our findings and the aforementioned literature regarding baseline LAVi may be attributed to the relatively small size of our study population and the relatively long duration from diagnosis to intervention in our study participants.

Limitations

Several limitations warrant consideration in interpreting our findings. Firstly, the relatively small size of our study population may limit the generalizability of our results to broader populations of AF patients. Additionally, the extended duration from AF diagnosis to intervention in our cohort might have introduced variability in baseline measurements, potentially influencing the observed echocardiographic outcomes. Furthermore, the absence of a control group undergoing an alternative treatment strategy, such as pharmacological rhythm control, restricts our ability to make direct comparisons and draw conclusions regarding the superiority of EC over other approaches. Moreover, the six-month follow-up duration may not capture longer-term changes in cardiac structure and function, and a more extended follow-up period could provide a more comprehensive understanding of the sustained effects of SR restoration.

## Conclusions

In the current study, we observed significant improvements in various cardiac parameters among patients maintaining sinus rhythm, such as LVEF, LVGLS, LAEF, LAVi, LVESVi, left atrial dimensions, mitral and tricuspid regurgitation severity, and symptoms severity, highlighting cardiac reverse remodeling after cardioversion. Notably, the study identifies cardiac output as a significant predictor of AF recurrence, emphasizing the importance of comprehensive assessments in predicting long-term outcomes.
